# Aging in smog: How air pollution and inequality shape relocation decisions among China's older adult

**DOI:** 10.3389/fpubh.2025.1589168

**Published:** 2025-06-11

**Authors:** Zhifei Xiong, Ju He, Chenchen Guo, Wenzhong Zhang

**Affiliations:** ^1^Key Laboratory of Regional Sustainable Development Modeling, Institute of Geographic Sciences and Natural Resources Research, Chinese Academy of Sciences, Beijng, China; ^2^College of Resources and Environment, University of Chinese Academy of Sciences, Beijing, China; ^3^Research Institute of Central Jiangsu Development, Yangzhou University, Yangzhou, China

**Keywords:** air pollution, satisfaction, relocation, older adults, China

## Abstract

**Objective:**

This study investigates the relationship between air pollution perceptions and relocation intentions among older adult populations in China. It aims to understand how dissatisfaction with air quality influences the willingness of older adult individuals to relocate from heavily polluted urban areas, while also examining the moderating effects of urban and individual characteristics.

**Methods:**

Utilizing data from official, confidential surveys from Urban Health Check Program in China in 2023, we employed a two-stage analytical framework to analyze the dual mechanisms through which air pollution impacts migration decisions: direct environmental push factors and perception-mediated psychological pathways. Regression analysis was conducted to quantify the mediation effects of urban characteristics and individual attributes on health risk perceptions among older adults.

**Results:**

The findings indicate that air quality dissatisfaction significantly predicts the willingness to relocate, especially in cities with high concentrations of pollutants like PM_2.5_, NO_2_, SO_2_, and CO. Air quality satisfaction was identified as a critical mediator in the relationship between pollutant levels and relocation intentions. Additionally, socio-economic disparities and intergenerational dynamics were found to complicate relocation decisions, with some older adult individuals developing attenuated pollution risk perceptions due to limited health literacy and adaptive resources.

**Conclusion:**

This study underscores the vital role of older adult individuals' perceptions in shaping their responses to air pollution and relocation intentions. It highlights the urgent need for targeted interventions that enhance health literacy, address environmental inequalities, and consider intergenerational dynamics in policy-making. Strategies such as health education programs, subsidized relocation initiatives, and supportive policies for caregivers are essential for fostering healthier living environments and improving the overall wellbeing of older adults amid ongoing environmental challenges.

## 1 Introduction

The great smog of 1952 claimed over 4,000 lives—predominantly older adult individuals. Air pollution has a significant negative impact on both the physical (1–3) and mental health (4–6) of citizens, with the older adult being particularly severely affected. Pollutants in the air, such as PM_2.5_, PM_10_, SO_2_, and NO_2_, have been found to adversely affect the sleep (7), lung (8) and cardiovascular health (9), and mental wellbeing (10, 11) of the older adult. Elevated concentrations of these pollutants correlate with increased mortality rates and reduced life expectancy in this population (12, 13). The use of air purifiers, wearing masks, and minimizing outdoor activities can effectively mitigate both the physical and psychological impacts of air pollution. However, these measures have less cost but make activity restriction which may increase the depression of older adults. None of them are as effective and permanent as relocating to an area with better air quality. As McLeman and Smit (14) mentioned, migration becomes part of the wider suite of potential responses by which populations vulnerable to particular climate-change impacts might adapt. Amenity-related factors have increasingly influenced the destination choices (15). Therefore, relocating to a better air environment has become a significant choice to the older adults.

Distinct from economically motivated mobility, environmental relocation among older adults manifests through three unique pathways: health-driven relocation, intergeneration escort migration and retirement destination shifts. The focus of this study is on health-driven relocation, which refers to the behavior of older adult individuals moving to environments with better air quality to meet their health needs. Intergenerational escort migration arises from the requests of older adults' children. This may stem from the children's desire to enhance their parents' living environment, or it may be driven by the need for their parents to assist in caring for their grandchildren (16). Retirement destination shifts, to a greater extent, provide older adult individuals with the opportunity to relocate to other cities which have better air condition.

However, living in an environment with better air quality may require individuals to pay a higher price. In cities where air quality is better, areas with lower pollution levels often have significantly higher housing prices compared to those with more severe air pollution (17–20). This economic disparity implies that older adult individuals—or their families—must navigate complex trade-offs between health priorities and financial constraints when choosing residential locations in varying air quality contexts This phenomenon also gives rise to various issues related to environmental injustice (21, 22). Low-income groups and groups who live in less developed regions are not only more vulnerable to exposure to air pollution but often lack representation in the policy-making process. Socioeconomic vulnerabilities, including lower income, limited education, and disparities in urban development, may exacerbate the health impacts of air pollution on older adults (23, 24). Additionally, the health repercussions of pollution can result in a gradual decline in residents' labor capacity and an increase in their medical expenses, further intensifying the inequalities associated with air pollution.

This assumption relies on a theoretical framework of perfect human rationality. By focusing on migration within the context of vulnerability and adaptation processes, the agency of actors can easily be obscured or overlooked (25). A divergence exists between public perceptions of air quality and objective measurements, mediated by socioeconomic status, environmental awareness, and psychological factors (26). In other words, individuals are affected by factors such as their education level and income, which limits their ability to objectively evaluate the impact of variations in air quality on their wellbeing. Particularly for the older adult, prolonged residence in a certain location leads to perceptual adaptation to the local air environment. Yet prolonged exposure may have already inflicted irreversible damage to their physiological health. This represents a form of implicit perceptual environmental inequality. Although numerous studies have analyzed the relationship between air pollution and the health of older adults, there is still a lack of discussion on whether older adults can perceive differences and change in air quality, how they experience these variations, the factors that influence this perceptual process, and the responses, such as relocating, they may exhibit. This study investigates these questions by analyzing urban air pollution levels in conjunction with older adults' perceptual adaptations and behavioral coping strategies.

China provides a critical empirical setting for this investigation due to its unique socio-environmental dynamics. In the past decade, air quality management has emerged as a critical political issue in China. Air pollution in China has been effectively mitigated through robust government interventions. While maintaining economic growth, China has seen a continuous improvement in air quality levels, with emissions of various air pollutants generally exhibiting a downward trend. However, China is also confronted with the challenge of profound aging and uneven development. This has positioned air quality as a key issue in the development of livability in Chinese cities, alongside concerns related to resident health and equality. Therefore, this study aims to analyze the relationship between urban air quality and the subjective perceptions of the older adults, as well as the mediating role of these perceptions in their behavioral responses to urban air pollution. Operationally, the study quantifies subjective perceptions through older adult residents' self-reported satisfaction with air quality in their current cities (2023 data), while behavioral responses are measured by relocation intentions over a 5-year horizon. Through this approach, the study seeks to uncover the implicit environmental inequality embedded in these perceptions.

## 2 Materials and methods

### 2.1 Relationship among air pollution, perception, and relocation of older adults

**Figure 1:**
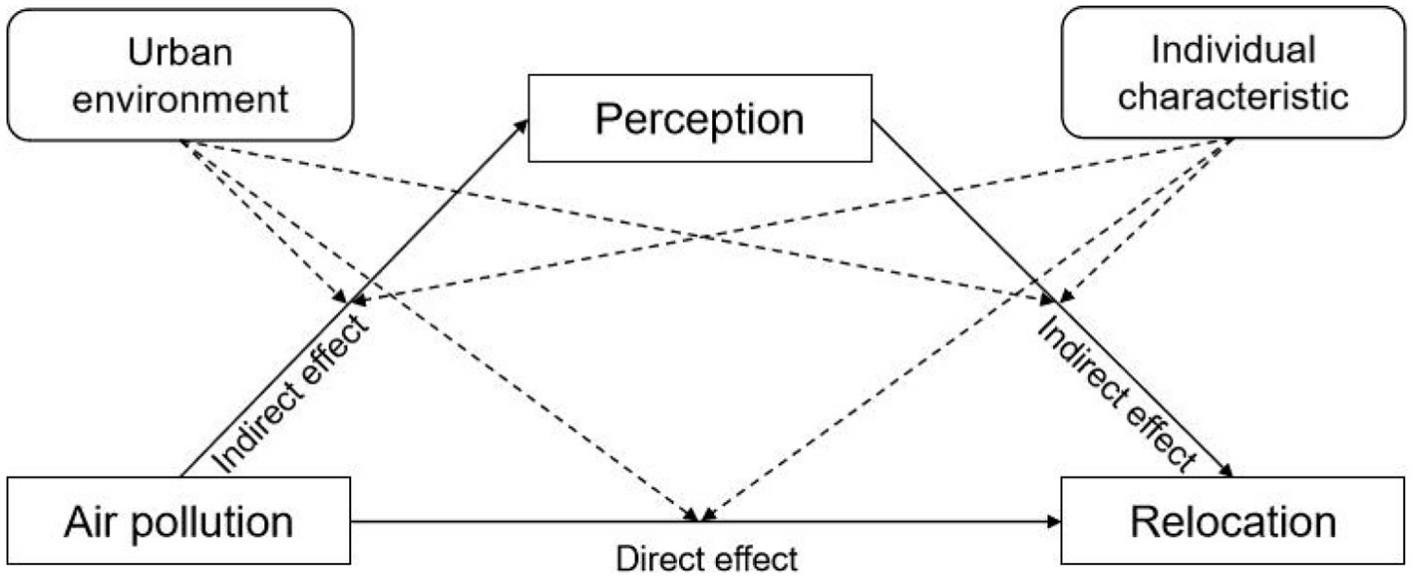
Perception of air pollution and relocation decision-making among the older adults.

Academic studies on environmental pollution have convincingly acknowledged the salient relevance of ambient pollutant emissions on individual life satisfaction (27). Empirical evidence suggests that air pollution erodes residential satisfaction through health deterioration mechanisms (28). Another issue is the relationship between air pollution and relocation. Relocation is a significant behavioral response of residents to air pollution. Relocating to low-pollution areas demonstrably improves health outcomes, particularly for vulnerable populations (29). Studies demonstrate the differential capitalization of air pollution risks in intercity housing markets, reflecting residents' willingness to pay for cleaner environments (30, 31). This means that residents are beginning to consider the impact of the air quality in their living environment and are willing to pay a corresponding price to relocate. Thus, pollution perception serves as a critical mediator linking environmental exposure to relocation decision-making. We have constructed a framework diagram to elucidate these two relationships. As shown in Figure 1, residents perceive the negative impact of air pollution on themselves, leading them to consider relocating to avoid these adverse effects.

The process of perceiving air quality and making relocation decisions is complex and unclear for older adult individuals. This study posits that there may be a bidirectional pathway of perception mediation. Under ideal conditions, older adults should undergo a process that follows the sequence of pollution exposure, leading to physiological or psychological harm, resulting in health stress, and ultimately forming the intention to relocate. A negative mediation pathway may also exist, where older adults are exposed to air pollution over the long term, leading to neural adaptation in the orbitofrontal cortex (32), resulting in desensitization of perception and suppression of the intention to relocate.

There are also other influencing factors in the relocation decision-making process, which include both urban-scale and individual-scale components. At the macro level, intercity pollution disparities stem from geographic heterogeneity and structural factors, including urbanization trajectories (33, 34) and industrial composition (35). On the other hand, differences at the individual scale can also result in varying probabilities of different groups being exposed to air pollution. Spatially concentrated vulnerabilities, where minority, older adult, and low-income communities disproportionately cluster in high-exposure zones, reflect systemic environmental injustice (36–40).

However, for older adults, certain factors play a more significant role in the relocation decision-making process. One of these factors is the emotional anchoring mechanism that older adult individuals employ when formulating their relocation plans. The environment can be an attitudinal and emotional context for older people to develop place attachment (41). In China, older adults often reside in one city for a long time. Relocating to another city means they have to give up the social network they have built over the years and confront the attachment to their local environment. Another emotional factor influencing this decision is the responsibility of caring for children, which is deeply rooted in Confucian cultural values in China. Grandparenting is particularly prevalent in China because of the increasing number of women in the labor force, shortages of daycare services, and intergenerational bonds (42). Once older adults' children have children of their own, it is highly likely that they will be asked by their children to relocate to the city where they live or to stay in the local area to help care for their grandchildren.

Another important issue is the household registration system (hukou). The household registration system is a significant factor limiting migration in China among cities. Having a non-local hukou means that residents will incur higher costs to purchase property and settle in that city, and they will not be able to enjoy many of the local policy benefits, such as access to local schools for children and healthcare insurance. Furthermore, as some studies have pointed out, air pollution is a significant factor contributing to the substantial loss of workforce productivity (43). Studies also indicate that older adult dependents of migrant workers are discouraged from migrating, while migrants growing old tend to return to the origins than to remain in the destinations (44).

Therefore, this study suggests that perception plays an important bidirectional mediating role in environmental migration among older adults. As an important vulnerable group, the physical frailty of the older adult may make them more sensitive to and dissatisfied with the air pollution in their living environment. However, this process is complex for the older adults. Sensory adaptation through prolonged exposure can attenuate older adult residents' awareness of pollution-induced health risks, despite accumulating physiological damage. This creates an age-related mobility gap that younger populations exhibit higher migration capacity than their older adult counterparts constrained by physiological and socioeconomic factors. In addition to the constraints on mobility imposed by the older adult individual's physical health and economic status, the adaptation process to a completely new environment can also generate significant stress for this demographic. This study therefore examines how multilevel moderators, spanning city characteristics and individual socioeconomic profiles, shape older adults' pollution perception and subsequent relocation behaviors.

### 2.2 Statistic method

Guided by migration push-pull theory, we operationalized relocation probability through two key determinants: (1) intercity air quality differentials as attraction forces, and (2) geographic distance as mobility friction. In this context, the difference in air quality has a positive effect, as residents are inclined to relocate to cities with better air quality. The influence of geographical distance, on the other hand, is negative. Therefore, the likelihood of migration among the older adult due to differences in air quality can be represented by the following indicator, as shown in Formula 1:


(1) $$\begin{array}{l} {\text{Probability}}_{{\text{air}}_{\text{i}}}=\frac{\sum_{\text{j}=1}^{\text{n}}\frac{\left({\text{Air}}_{\text{i}}-{\text{Air}}_{\text{j}}\right)}{{\text{d}}_{\text{ij}}}}{\text{n}} \end{array}$$

Where Probability_ec_o__i__ is the probability of older adults in city i relocate due to economics levels. Eco_i_ and Eco_j_ are the economics levels of city i and city j. And N is the number of all cities in China.

And we used an ordinary least square regression model to test the impact of air pollution on satisfaction. It took the following functional form:


(2) $$\begin{array}{l} \text{SAT}={\text{β}}_{0}+{\text{β}}_{1}\text{pollutants}+\text{μ} \end{array}$$

Where SAT is the residents' satisfaction level on air pollution, while pollutants represent the air pollution index including concentration of PM_2.5_, PM_10_, NO_2_, SO_2_, CO, and O_3_ of cities where residents live in.

However, the disparities on satisfaction may come from the diversity of development of cities, household composition, income or housing types. These factors may have significant controlling and moderating effects on residents' perception of air quality. Thus, we introduced the demographic characteristics and housing types of respondents in Models 3 and 4:


(3) $$\begin{array}{l} \text{SAT}={\text{β}}_{0}+{\text{β}}_{1}\text{pollutants}+{\text{β}}_{2}\text{city}+{\text{β}}_{3}\text{residents}+\text{μ} \end{array}$$

(4) $$\begin{array}{l} \text{SAT}={\text{β}}_{0}+{\text{β}}_{1}\text{pollutants}+{\text{β}}_{2}\text{city}+{\text{β}}_{3}\text{residents}\\ \;+{\text{β}}_{4}\text{pollutants}\times \text{city}+{\text{β}}_{5}\text{pollutants}\times \text{residents}+\text{μ} \end{array}$$

*city* represents the socioeconomic attributes of the city where residents live, such as the decline rate in urban pollutant concentrations over the past decade (hereinafter referred to as the pollutants concentration decline rate), gross domestic product (hereinafter referred to as GDP), population and the ratio of housing prices to residents' annual disposable income. *Residents* signifies the individual characteristics of residents, including gender, income, health status, and others, which can be referred to Table 1 for specifics.

**Table 1:** Descriptive statistics of survey.

**Indicators**	**Alternatives**	**Ratio**	**Mean**
Satisfaction	Very satisfied	39.22%	
	Satisfied	35.85%	
	Neutral	16.21%	
	Dissatisfied	7.29%	
	Very dissatisfied	1.42%	
Relocate	Yes	8.25%	
	No	91.75%	
Property	Ownership	86.94%	
	Rental	13.06%	
Living time	< 5 years	16.37%	
	5–10 years	19.20%	
	More than 10 years	64.43%	
Gender	Male	49.21%	
	Female	50.79%	
Employment status	Employed	20.40%	
	Unemployed (including the retired)	79.60%	
Household income (per year)	< 50,000 yuan	34.63%	
	50,000–100,000 yuan	36.648%	
	More than 100,000 yuan	28.89%	
Relative income	Higher	13.60%	
	Nearly	30.68%	
	Lower	55.96%	
Hukou (household registration)	Local	76.50%	
	Non-local	23.50%	
Health	Healthy	14.30%	
	Relatively healthy	32.60%	
	Neutral	46.30%	
	Relatively unhealthy	5.45%	
	Unhealthy	1.25%	
Living alone or residing with a spouse	Yes	73.31%	
	No	26.69	
Exercise times (per week)			2.52

This study posits that there exists a similar relationship between residents' satisfaction with air quality and their decision to relocate. Given that the willingness to relocate is a binary variable, a logistic regression model is employed for the analysis.


(5) $$\begin{array}{l} \text{P }\left(\text{Relocate}=1|\text{SAT}\right)=\frac{1}{1+e^{-\left({\text{β}}_{0}+{\text{β}}_{1}\text{SAT}\right)}} \end{array}$$

(6) $$\begin{array}{l} \text{P }\left(\text{Relocate}=1|\text{SAT},\text{ city},\text{ resident}\right)\;\\ =\frac{1}{1+e^{-\left({\text{β}}_{0}\;+{\text{β}}_{1}\text{ SAT }+\;{\text{β}}_{2}\text{city }+\;{\text{β}}_{3}\text{residents}\right)}} \end{array}$$

(7) $$\begin{array}{l} \text{P }\left(\text{Relocate}=1|\text{SAT},\text{ city},\text{ resident},\text{ SAT }\times \text{city},\text{ SAT }\times \text{ residents}\right)\\ \;=\frac{1}{1+e^{-({\text{β}}_{0}+{\text{β}}_{1}\text{SAT }+{\text{β}}_{2}\text{ city }+\;{\text{β}}_{3}\text{ residents}+\;{\text{β}}_{4}\text{SAT }\times \text{ city }+\;{\text{β}}_{5}\text{ SAT }\times \text{ residents})}} \end{array}$$

Due to the involvement of decision-making regarding the relocation of older adults and the avoidance pf multicollinearity issues, we replace indicators concentration of pollutants and GDP with Probability_air_ and Probability_eco_ at the urban scale.

The above Models 2–7 collectively constitute the indirect effects in the mediating effect of air pollution–older adult perception–relocation decision. The total effect formed by the relocation decision of the older adult and the local air quality is composed of Models 8–10:


(8) $$\begin{array}{l} \text{P }\left(\text{Relocate}=1|\text{pollutants}\right)=\frac{1}{1+{\text{e}}^{-\left({\text{β}}_{0}+{\text{β}}_{1}\text{pollutants}\right)}} \end{array}$$

(9) $$\begin{array}{l} \text{P }\left(\text{Relocate}=1|\text{pollutants},\text{city},\text{resident}\right)\\ \;=\frac{1}{1+{\text{e}}^{-\left({\text{β}}_{0}+{\text{β}}_{1}\text{pollutants}+{\text{β}}_{2}\text{city}+{\text{β}}_{3}\text{residents}\right)}} \end{array}$$

(10) $$\begin{array}{l} \text{P }\left(\text{Relocate}=1|\text{pollutants},\text{city},\text{resident},\text{pollutants}\times \text{city},\text{pollutants}\times \text{residents}\right)\\ =\frac{1}{1+{\text{e}}^{-\left({\text{β}}_{0}+{\text{β}}_{1}\text{pollutants}+{\text{β}}_{2}\text{city}+{\text{β}}_{3}\text{residents}+{\text{β}}_{4}\text{pollutants}\times \text{city}+{\text{β}}_{5}\text{pollutants}\times \text{residents}\right)}} \end{array}$$

The control variables and moderating variables here are consistent with those in Models 6–8. Based on the determination of total effects and indirect effects, this study will use the bootstrap method to validate the mediating effects. Additionally, given that certain indicators in this study pertain to two levels of analysis—cities and individual older adult persons—the overall satisfaction of older adult residents in each city regarding air quality is represented by the average satisfaction score of the surveyed individuals. Meanwhile, the willingness of older adults in each city to relocate is quantified by the probability of those who expressed a willingness to move to the total number of respondents surveyed.

### 2.3 Data

In the empirical analysis, data can be classified into three parts. Firstly, we utilized data from the resident survey conducted as part of the Urban Health Check Program in China. This program targeted local residents and employed electronic questionnaires to assess their satisfaction with urban development and policies, as well as their own socio-economic attributes. Led by the Ministry of Housing and Urban-Rural Development of China, the survey was conducted from March to September 2023 across 58 municipalities and prefecture-level cities in China (the questionnaires were primarily distributed in August and September). To enhance the quality of the survey, the Ministry organized professional personnel to conduct project guidance workshops at different administrative levels, ensuring that staff at all levels fully understood the objectives of the program and the use of the electronic questionnaires. Ultimately, this was implemented at the community level, with street administrative personnel visiting each respondent to assist them in completing the electronic questionnaire.

**Figure 2:**
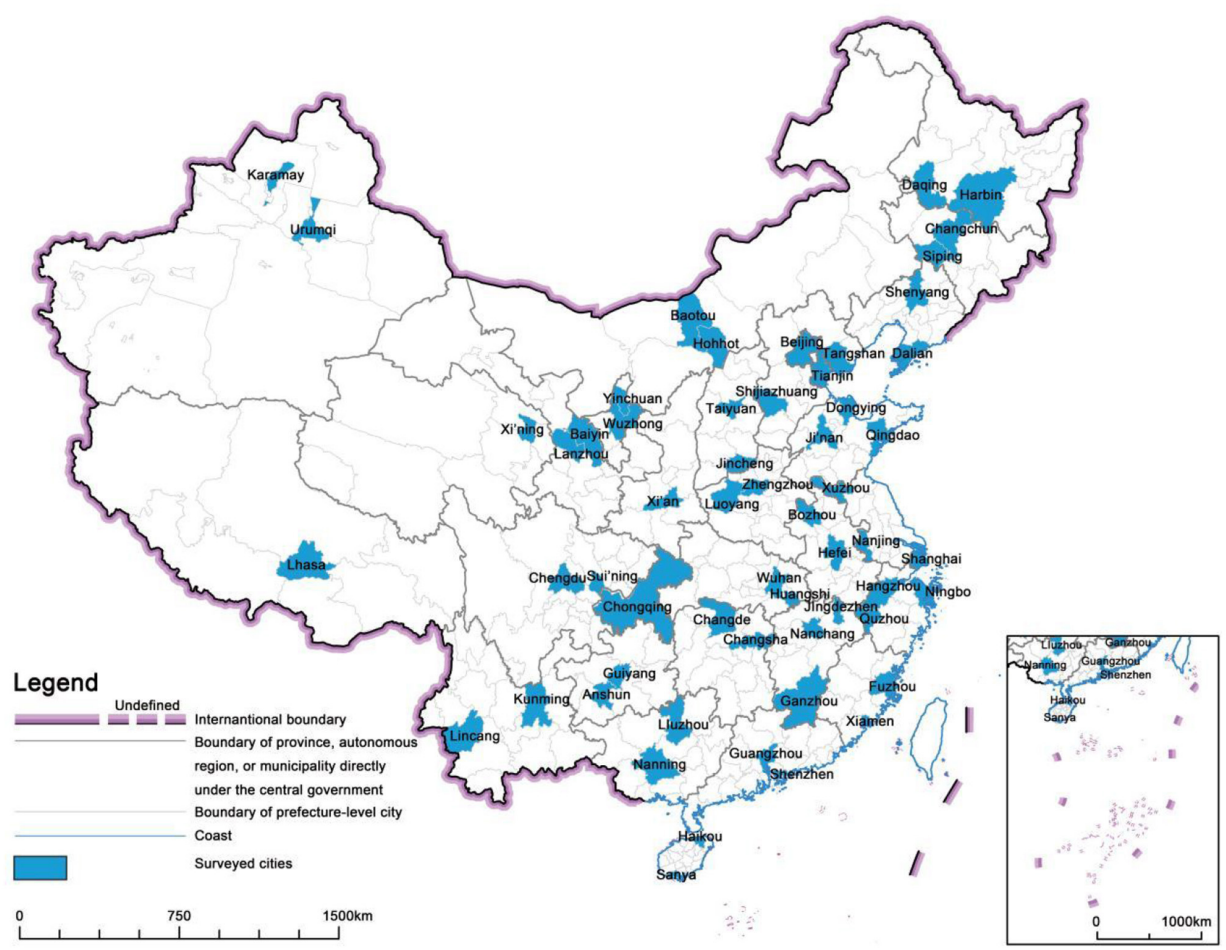
Distribution of surveyed cities.

In this study, a total of 42,927 questionnaires from individuals aged 60 and above were utilized as the research sample. The satisfaction assessment of air quality in this survey questionnaire used a Likert five-point scale for responses, ranging from “very satisfied, satisfied, neutral, dissatisfied, very dissatisfied” in order of preference. The relevant questions from the questionnaire and the corresponding statistical results are presented in Table 1. And the specific questionnaire items are included in the Appendix 1. Furthermore, although the questionnaire only encompasses 58 cities, these cities are relatively evenly distributed across China's 31 provinces (excluding Hong Kong, Macao, and Taiwan), encompassing diverse population sizes and economic scales. This approach mitigates potential biases in data analysis arising from the selection of city samples. Figure 2 shows the distribution of these 58 cities.

Secondly, air pollution metrics for cities, including concentration of PM_2.5_, PM_10_, NO_2_, SO_2_, CO, and O_3_ in 2023 are obtained from China Air Quality Online Monitoring and Analysis Platform (https://www.aqistudy.cn/historydata). These six types of pollutants are common indicators for measuring air pollution. Although they exhibit certain correlations in spatial distribution, their health effect pathways are significantly different. Fine particulate matter penetrates the alveoli and enters the bloodstream, directly triggering systemic inflammation. NO_2_ and SO_2_ primarily irritate the respiratory mucosa, exacerbating asthma and chronic obstructive pulmonary disease. O_3_ damages lung function through oxidative stress, while CO causes tissue hypoxia by binding to hemoglobin, leading to cardiovascular events. These differences in biological mechanisms may result in significant variations in older adult individuals' sensitivity to various pollutants and their level of health concerns, subsequently affecting their satisfaction assessments. Therefore, in this study, the concentrations of these six pollutants are treated as independent variables. At last, city social-economic data such as GDP and population is come from 2023 China City Statistic Yearbook.

## 3 Distribution of air pollution, satisfaction, and relocation decision-making

**Figure 3:**
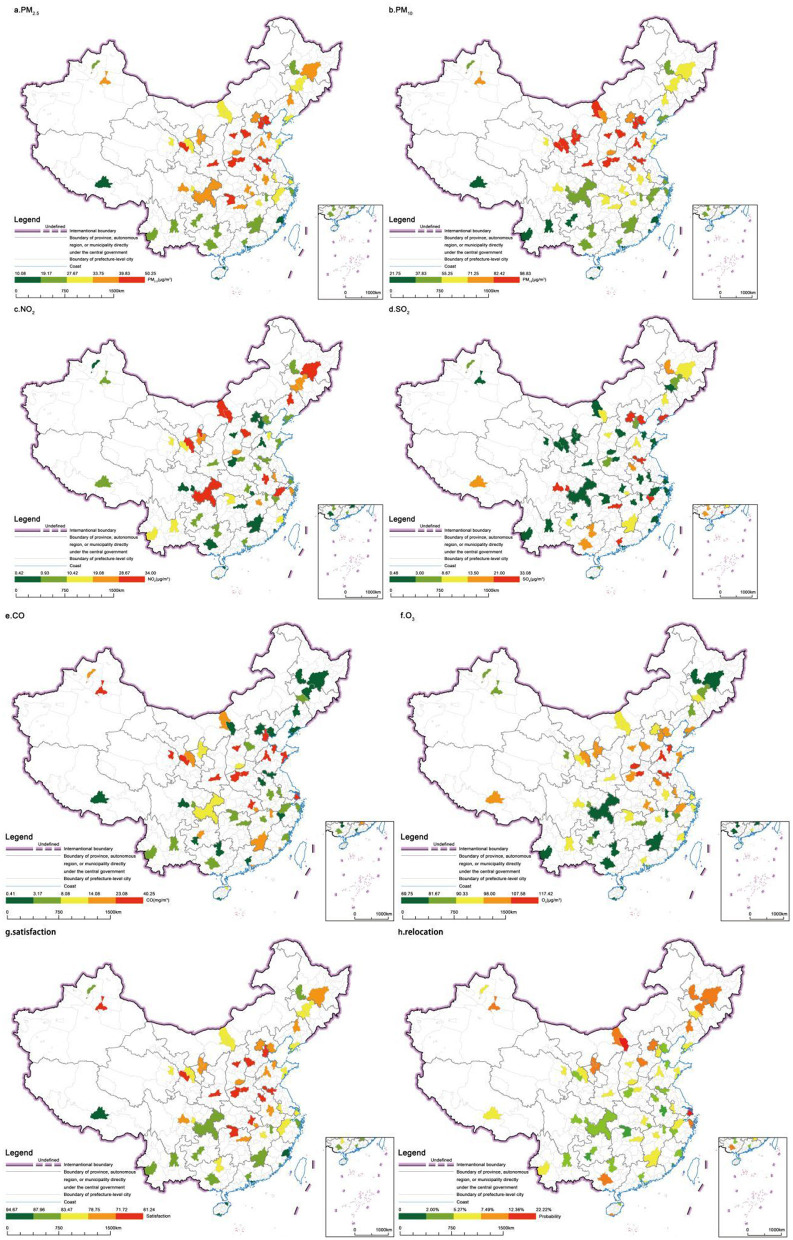
Distribution of air pollution, satisfaction, and decision-making to relocating in China.

We constructed city-level indices for satisfaction and relocation willingness by calculating the average satisfaction level of surveyed older adults in each city and the proportion of older adults planning to relocate within the next 5 years relative to the total number of older adult respondents in that city. Figure 3 illustrates the distribution of air pollution across Chinese prefecture-level cities and the corresponding satisfaction levels of local residents regarding the air quality in their living environments. PM_2.5_, PM_10_, and O_3_ concentrations exhibit a latitudinal gradient, increasing consistently from southern to northern China. A significant accumulation of fine particulate matter and ozone is concentrated over the North China Plain. China's heating policy necessitates the burning of substantial amounts of coal in the northern regions during winter. The considerable diurnal temperature fluctuations and dry climate hinder the settling of fine particulate matter emitted from combustion into the atmosphere. Furthermore, the concentration of the population and the presence of heavy industry amplify this issue. Over the past decade, nitrogen dioxide, sulfur dioxide, and carbon monoxide have also been predominantly concentrated in this region (45–48). These pollutants primarily arise from the combustion of fossil fuels and vehicle emissions. However, with the recent tightening of regulations on industrial emissions and the shift toward new energy vehicles, this concentration phenomenon has become significantly less pronounced.

**Figure 4:**
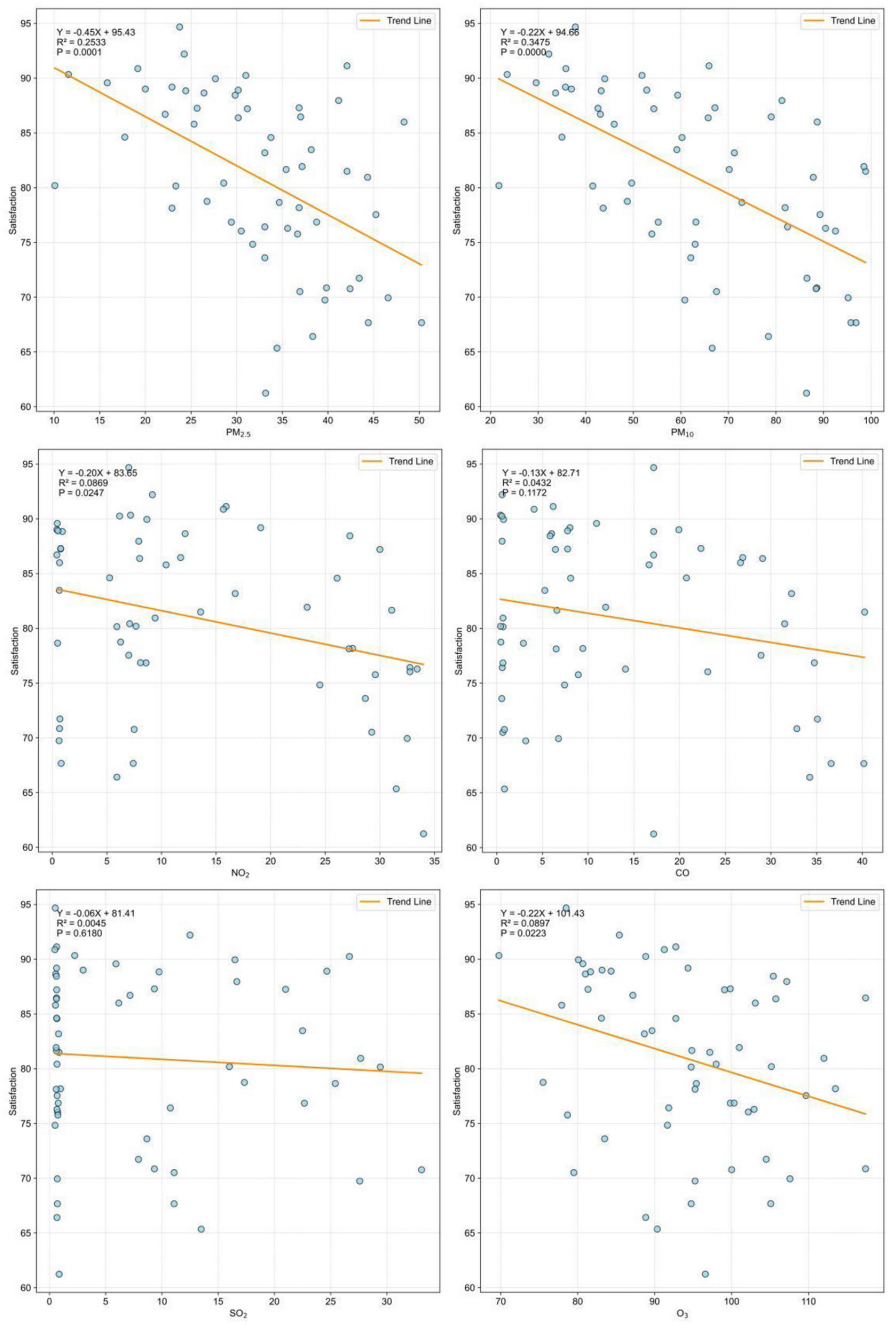
Correlation between air pollution and satisfaction by cities.

The satisfaction levels of older adults in China concerning air quality exhibit a spatial distribution trend that declines from south to north. Figure 4 further demonstrates the correlation between resident satisfaction and the spatial distribution patterns of air pollution. Among the six pollution indicators, only PM_2.5_ (*p* < 0.01), PM_10_ (*p* < 0.01), NO_2_ (*p* < 0.05), and O_3_ (*p* < 0.05) showed statistically significant correlations in the hypothesis tests. With the increase in pollutant concentrations, the satisfaction levels of local residents exhibit a clear declining trend. PM_2.5_, and PM_10_ exhibited stronger explanatory power, with adjusted *R*^2^ values ranging from 20% to over 30% in the regression models, while NO_2_ and O_3_ also passed the hypothesis testing, their explanatory power remains below 10%.

**Figure 5:**
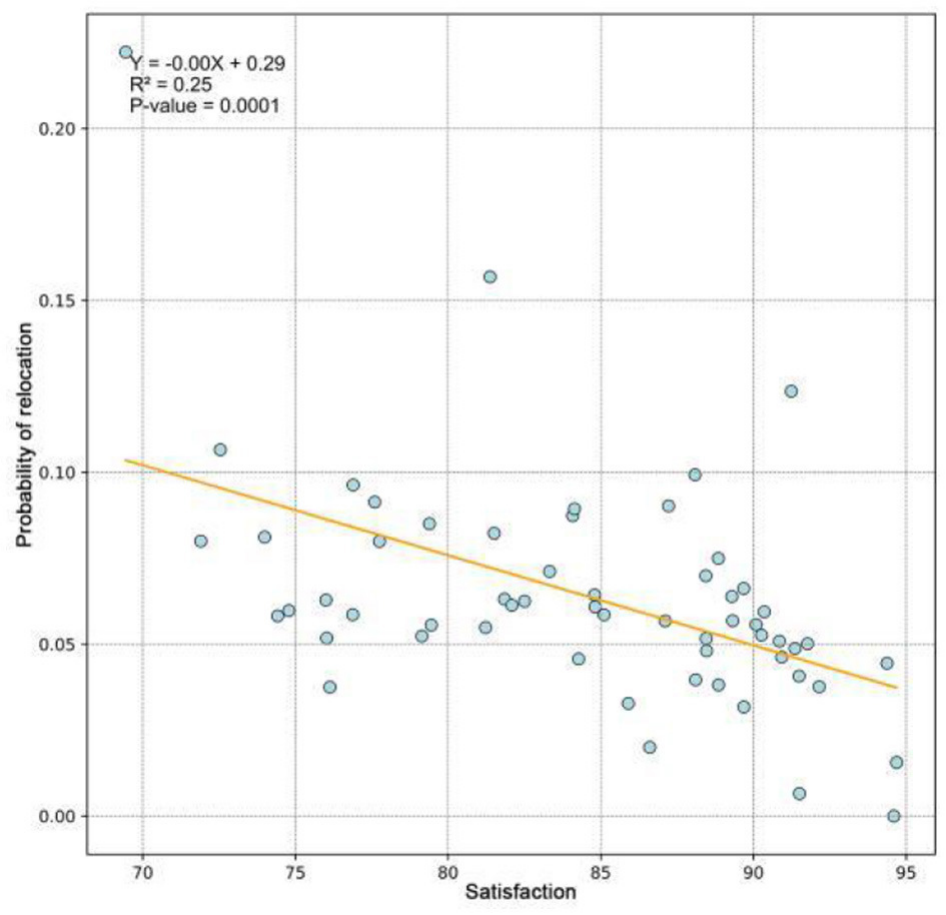
Correlation between probability of relocation and satisfaction by cities.

The willingness of older adults to relocate exhibits a distinct spatial pattern, demonstrating a higher propensity in northern regions compared to their southern counterparts. Figure 5 further illustrates that in cities characterized by lower overall satisfaction with air quality among older adult residents, there is a correspondingly stronger inclination among these individuals to consider relocation. Notably, cities with higher air quality satisfaction scores, such as Shanghai, Ningbo, and Sanya, still exhibited elevated relocation willingness. This paradox may be attributed to relatively high economic development levels within China. Higher urbanization levels correlate with increased cost of living, potentially offsetting the welfare benefits of air quality improvements for older adult populations.

## 4 Regression results

### 4.1 Mediation effect test

We performed a mediation analysis using the Bootstrap method with 5,000 resamples (95% CI), and the results are summarized in Table 2. Except for O_3_, the total effects of all pollutant concentrations on the willingness of older adults to relocate passed the significance test. Furthermore, with the exception of PM_2.5_, the concentrations of four other pollutants showed a statistically significant positive correlation with the willingness of older adult individuals to relocate, as evidenced by confidence intervals excluding zero. This means that, in terms of total effects, as PM_10_ and the concentrations of NO_2_, SO_2_, and CO in the city increase, older adults are more likely to have a higher willingness to relocate, whereas PM_2.5_ has the opposite effect.

**Table 2:** Mediation effect detection of older adults satisfaction based on bootstrap method.

**Independent variable**	**Effect type**	**Coefficient**	**Standard error**	**95% CI lower**	**95% CI upper**
PM_2.5_	Total effect	−0.004	0.004	−0.005	−0.004
	Direct effect	−0.005	0.000	−0.006	−0.004
	Indirect effect	0.001	0.000	0.011	0.034
	Mediation types	Masking effect			
PM_10_	Total effect	0.001	0.000	0.000	0.001
	Direct effect	0.000	0.000	0.000	0.001
	Indirect effect	0.000	0.005	−0.012	0.015
	Mediation types	The mediation effects are not significant			
NO_2_	Total effect	0.001	0.000	0.000	0.001
	Direct effect	−0.001	0.000	−0.001	0.000
	Indirect effect	0.002	0.004	0.062	0.080
	Mediation types	Masking effect			
SO_2_	Total effect	0.001	0.000	0.001	0.002
	Direct effect	−0.000	0.000	−0.001	0.000
	Indirect effect	0.002	0.004	0.068	0.085
	Mediation types	Full mediation effect			
CO	Total effect	0.001	0.000	0.000	0.002
	Direct effect	−0.001	0.000	−0.001	0.000
	Indirect effect	0.002	−0.005	0.065	0.083
	Mediation types	Full mediation effect			
O_3_	Total effect	0.000	0.000	−0.000	0.001
	Direct effect	0.000	0.000	−0.000	0.001
	Indirect effect	0.000	0.002	−0.000	0.008
	Mediation types	The mediation effects are not significant			

0.000 is the smaller regression result with three decimal places retained.

At the same time, for PM_2.5_, NO_2_, and CO, although their direct effects differ, the indirect effects caused by older adults' satisfaction with air quality are all positive. This means that for these three types of air pollutants, the older adult's perception of air quality somewhat enhances their willingness to relocate. Even if the concentration of certain pollutants (such as PM_2.5_ and NO_2_) increases, potentially leading to direct negative effects, the older adult's perception of air quality can somewhat alter this relationship, enhancing the positive correlation between the increase in air pollutant concentrations and the willingness of older adult individuals to relocate.

Notably, despite non-significant mediation pathways for PM_10_ and O_3_, their direct effects suggest potential unmeasured mechanisms that could independently influence relocation decisions, warranting further investigation.

### 4.2 Moderating effect test

Table 3 summarizes the multivariate linear regression results examining the association between air quality indicators and satisfaction levels. In Model 2, all pollutant concentration metrics (PM_2.5_, PM_10_, NO_2_, SO_2_, CO, and O_3_) showed statistically significant negative coefficients, indicating that older adults' satisfaction decreases systematically with deteriorating air quality.

**Table 3:** Results of regression between satisfaction and air pollution.

**Variable**	**PM**_**2.5**_			**PM**_**10**_			**NO**_**2**_			**SO**_**2**_			**CO**			**O**_**3**_		
	**Model (2)**	**Model (3)**	**Model (4)**	**Model (2)**	**Model (3)**	**Model (4)**	**Model (2)**	**Model (3)**	**Model (4)**	**Model (2)**	**Model (3)**	**Model (4)**	**Model (2)**	**Model (3)**	**Model (4)**	**Model (2)**	**Model (3)**	**Model (4)**
Pollutants concentration	−0.020^***^	−0.012^***^	−0.010^***^	−0.009^***^	−0.008^***^	−0.001	−0.002^***^	−0.005^***^	−0.012^***^	−0.004^***^	−0.008^***^	−0.007^***^	−0.006^***^	−0.005^***^	−0.004^**^	−0.010^***^	−0.018^***^	−0.010^***^
Pollutants concentration decline rate		−0.071^***^	−0.096^***^		−0.029^***^	0.009		−0.013^***^	0.018^***^		−0.113^***^	−0.111^***^		−0.009	0.047^***^		−0.072^***^	−0.652^***^
Population		−0.000^***^	0.001^***^		−0.000^***^	0.001^***^		−0.000^***^	−0.000^***^		−0.000^***^	−0.000^***^		−0.000^***^	0.000		−0.000^***^	0.001^***^
GDP		0.000	−0.000^***^		−0.000^***^	−0.000^***^		0.000^***^	0.000^***^		0.000^**^	−0.000^***^		0.000	−0.000^***^		0.000^***^	−0.000^***^
Housing price/income		−0.201^***^	1.630^***^		−0.432^***^	0.273		−0.621^***^	0.433^***^		−0.507^***^	0.828^***^		−0.731^***^	1.022^***^		0.049	1.382^**^
Property (rental)		−0.083^***^	−0.149^***^		−0.078^***^	−0.121^***^		−0.084^***^	−0.090^***^		−0.071^***^	−0.058^***^		−0.078^***^	−0.102^***^		−0.082^***^	−0.023
Living time (5–10 years)		−0.025	0.034		−0.024	0.067		−0.026^*^	−0.054^**^		−0.027^*^	0.004		−0.027^*^	−0.047^**^		−0.026^*^	0.268^**^
Living time (more than 10 years)		−0.025^*^	0.001		−0.023^*^	0.037		−0.019	−0.029		−0.018	0.004		−0.029^**^	−0.051^***^		−0.024^*^	0.142
Gender (female)		−0.059^***^	−0.043		−0.062^***^	−0.058^**^		−0.064^***^	−0.066^***^		−0.067^***^	−0.063^***^		−0.065^***^	−0.070^***^		−0.060^***^	−0.018
Employment status (unemployed)		0.061^***^	0.105^**^		0.061^***^	0.109^***^		0.074^***^	0.053^***^		0.065^***^	0.056^***^		0.076^***^	0.100^***^		0.061^***^	0.215^**^
Income (50,000–100,000 yuan per year)		0.024^**^	0.075		0.023^**^	0.073^*^		0.021^*^	0.020		0.026^**^	0.023		0.031^***^	0.047^***^		0.035^***^	0.327^***^
Income (more than 100,000 yuan per year)		−0.034^***^	0.120^**^		−0.036^***^	0.069^*^		−0.027^**^	−0.022		−0.021	−0.033^**^		−0.017	0.024		−0.008	0.361^***^
Hukou (non-local)		0.003	0.142^***^		0.005	0.143^***^		0.016	0.007		0.005	0.019		0.013	0.020		−0.017	0.135
Health (relatively healthy)		−0.172^***^	−0.075		−0.172^***^	−0.107^**^		−0.163^***^	−0.135^***^		−0.163^***^	−0.177^***^		−0.159^***^	−0.155^***^		−0.161^***^	−0.115
Health (neutral)		−0.353^***^	−0.144^***^		−0.352^***^	−0.213^***^		−0.345^***^	−0.315^***^		−0.344^***^	−0.350^***^		−0.339^***^	−0.332^***^		−0.344^***^	−0.154
Health (relatively unhealthy)		−0.485^***^	−0.302^***^		−0.484^***^	−0.318^***^		−0.475^***^	−0.452^***^		−0.480^***^	−0.505^***^		−0.472^***^	−0.433^***^		−0.481^***^	−0.085
Health (unhealthy)		−0.621^***^	−0.581^***^		−0.620^***^	−0.471^***^		−0.620^***^	−0.751^***^		−0.618^***^	−0.553^***^		−0.616^***^	−0.581^***^		−0.617^***^	0.226
Relative income (nearly)		−0.032^**^	−0.065		−0.030^**^	−0.011		−0.034^**^	−0.012		−0.029^*^	−0.021		−0.028^*^	−0.049^**^		−0.037^**^	0.164
Relative income (lower)		0.135^***^	−0.073		0.135^***^	−0.012		0.133^***^	0.145^***^		0.133^***^	0.125^***^		0.135^***^	0.106^***^		0.135^***^	0.099
Living condition (along or with spouse)		0.124^***^	−0.092^*^		0.136^***^	0.196^***^		0.201^***^	0.146^***^		0.164^***^	0.138^***^		0.216^***^	0.263^***^		0.139^***^	−0.685^***^
Exercise times		−0.000	0.017		0.000	0.027^*^		−0.001	−0.011^*^		−0.000	−0.004		−0.003	0.011^*^		0.001	0.124^***^
Pollutants × pollutants concentration decline rate			0.000			−0.000^**^			−0.006^***^			−0.001^**^			−0.005^***^			−0.006^***^
Pollutants × population			−0.000^***^			−0.000^***^			0.000^**^			−0.000^***^			−0.000^***^			−0.000^***^
Pollutants × GDP			0.000^***^			0.000^***^			0.000			0.000^***^			0.000^***^			0.000^***^
Pollutants × housing price/income			−0.052^***^			0.009^**^			0.034^***^			−0.063^***^			−0.040^***^			−0.010
Pollutants × property (rental)			0.002			0.001			0.001			−0.002			0.002^*^			−0.001
Pollutants × living time (5–10 years)			−0.002			−0.001^*^			0.002			−0.004^***^			0.001			−0.003^**^
Pollutants × living time (more than 10 years)			−0.001			−0.001			0.001			−0.003^***^			0.002			−0.002
Pollutants × gender (female)			−0.000			−0.000			0.000			−0.000			0.000			−0.000
Pollutants × employment (unemployed)			−0.001			−0.001			0.001			0.001			−0.002^**^			−0.002
Pollutants × income (50,000–100,000 yuan per year)			−0.001			−0.001			−0.000			0.000			−0.001			−0.003^***^
	**Model (2)**	**Model (3)**	**Model (4)**	**Model (2)**	**Model (3)**	**Model (4)**	**Model (2)**	**Model (3)**	**Model (4)**	**Model (2)**	**Model (3)**	**Model (4)**	**Model (2)**	**Model (3)**	**Model (4)**	**Model (2)**	**Model (3)**	**Model (4)**
Pollutants × income (more than 100,000 yuan per year)			−0.004^***^			−0.002^***^			−0.001			0.001			−0.003^***^			−0.004^***^
Pollutants × hukou (non-local)			−0.004^***^			−0.002^***^			0.001			−0.002^*^			−0.001			−0.002
Pollutants × health (relatively healthy)			−0.003			−0.001			−0.002^*^			0.001			−0.000			−0.000
Pollutants × health (neutral)			−0.006^***^			−0.002^***^			−0.002^**^			0.000			−0.000			−0.002
Pollutants × health (relatively unhealthy)			−0.005^**^			−0.003^**^			−0.002			0.002			−0.002			−0.004^*^
Pollutants × health (unhealthy)			−0.001			−0.002			0.011^***^			−0.006^*^			−0.002			−0.009^**^
Pollutants × relative income (nearly)			0.001			−0.000			−0.002			−0.001			0.001			−0.002
Pollutants × relative income (lower)			0.006^***^			0.002^***^			−0.001			0.001			0.002^*^			0.000
Pollutants × living condition (along or with spouse)			0.005^***^			−0.001^**^			0.002^*^			0.001			−0.002^**^			0.008^***^
Pollutants × exercise times			−0.000			−0.000^*^			0.001^**^			0.000			−0.001^***^			−0.001^***^
Constant	4.715^***^	4.796^***^	4.794^***^	4.606^***^	4.797^***^	4.380^***^	4.013^***^	4.056^***^	4.116^***^	4.083^***^	4.227^***^	4.282^***^	4.124^***^	4.176^***^	4.092^***^	5.003^***^	5.885^***^	5.145^***^

^***^p < 0.01; ^**^p < 0.05; ^*^p < 0.1.

After introducing control variables in Model 3, the various pollutant concentration indicators continued to exhibit a negative correlation with the satisfaction. At the urban scale indexes, all pollutants, except for CO, the decline rate of pollutants concentration shows a negative correlation with satisfaction levels. There is also a negative relationship between the population size in cities, the ratio of housing prices to disposable income and satisfaction, while the city's GDP is positively correlated with satisfaction. At the individual level, older adult individuals who are renting, have a shorter residence duration, are male, unemployed, have lower income and relative income, are in better health, or live alone or with a spouse tend to have higher satisfaction levels.

When interaction terms are introduced in Model 4, the significant negative correlation between the pollutants concentration and satisfaction remains, indicating that the older adults are aware of the negative impacts of air pollution. In terms of moderating effects, at the urban scale, the decrease rates of PM_10_, NO_2_, SO_2_, CO, and O_3_ concentrations negatively moderate the older adult's perception of air quality. Given that China has significantly intensified efforts to control air pollution in recent years, cities with higher rates of pollutant concentration reduction often also have higher baseline concentrations of these pollutants. Thus, the rate of decrease in pollutant concentrations reflects the overall pollution levels in those areas. Furthermore, the older adults tend to have resided in these cities for many years, meaning that air pollution has already had a considerable impact on them. The negative moderating effect of population size and the ratio of housing prices to disposable income indicate that even with comparable objective pollution levels, the psychological pressure resulting from overcrowding and high housing prices may amplify older adults' sensitivity to air pollution. A larger population typically indicates increased industrial activity and more crowded urban environments. Higher housing prices may also reduce the quality of the living environment for older adults. In contrast, GDP have positive moderating effects. At higher economic levels, the older adult may find it easier to access resources and support to improve their quality of life, thereby reducing their sensitivity to the negative impacts of air quality.

At the individual level, while the significance of the moderating effects of various variables differs across different pollutants, their negative impacts remain consistent. For instance, older adults with longer local residency, higher income and relative income, poorer physical health, and those living with non-spousal companions all exhibit attributes that, through moderating effects, diminish their satisfaction with air quality.

Table 4 presents the logistic regression results analyzing how air quality perceptions and contextual factors jointly shape older adults' relocation willingness. Three nested models (Models 5–7) progressively introduce mediators, moderators, and interaction terms to disentangle direct and moderated pathways. In Model 5, air quality satisfaction showed robust negative associations with relocation willingness, suggesting that as the satisfaction of older adult individuals with air quality declines, the likelihood of relocation can increase from 2.95 to 43%.

**Table 4:** Results of regression between decision–making of relocation and satisfaction.

**Variable**	**PM**_**2.5**_			**PM**_**10**_			**NO**_**2**_			**SO**_**2**_			**CO**			**O**_**3**_		
	**Model (5)**	**Model (6)**	**Model (7)**	**Model (5)**	**Model (6)**	**Model (7)**	**Model (5)**	**Model (6)**	**Model (7)**	**Model (5)**	**Model (6)**	**Model (7)**	**Model (5)**	**Model (6)**	**Model (7)**	**Model (5)**	**Model (6)**	**Model (7)**
Satisfaction	−0.802^***^	−0.907^***^	−1.010^***^	−0.802^***^	−0.899^***^	−0.804^***^	−0.802^***^	−0.871^***^	−0.240^**^	−0.802^***^	−0.892^***^	−0.371^***^	−0.802^***^	−0.892^***^	−0.440^***^	−0.802^***^	−0.875^***^	−0.266^***^
Probability_air_		−16.342^***^	−9.422		−4.221^***^	−6.001		−5.621	−23.982		−12.012	−22.479		−352.216	−1108.274		3.025	−15.471
Pollutants concentration decline rate		−0.207^***^	−1.004^***^		−0.153^***^	−0.659^***^		−0.054^***^	−0.085^*^		−0.112^***^	−0.268^***^		−0.277^***^	−1.189^***^		−0.229^***^	−0.411^***^
Population		−0.001^***^	−0.004^***^		−0.001^***^	−0.003^***^		−0.001^***^	−0.003^***^		−0.001^***^	−0.003^***^		−0.001^***^	−0.003^***^		−0.001^***^	−0.002^***^
GDP		0.000^***^	0.000^***^		0.000^***^	0.000^***^		0.000^***^	0.000^***^		0.000^***^	0.000^***^		0.000^***^	0.000^***^		0.000^***^	0.000^***^
Housing price/income		−0.907^***^	1.926^**^		−0.558^**^	3.526^***^		−0.020	5.429^***^		−0.564^**^	4.697^***^		0.077	5.355^***^		0.074	5.529^***^
Property (rental)		0.638^***^	−0.504^***^		0.632^***^	−0.510^***^		0.654^***^	−0.405^**^		0.666^***^	−0.380^**^		0.651^***^	−0.434^**^		0.675^***^	−0.379^**^
Living time (5–10 years)		−0.484^***^	0.759^***^		−0.482^***^	0.739^***^		−0.481^***^	0.643^***^		−0.480^***^	0.677^***^		−0.479^***^	0.725^***^		−0.481^***^	0.649^***^
Living time (more than 10 years)		−0.341^***^	0.421^**^		−0.344^***^	0.399^**^		−0.321^***^	0.425^**^		−0.297^***^	0.515^***^		−0.351^***^	0.307^*^		−0.323^***^	0.402^**^
Gender (female)		−0.006	0.161		−0.001	0.155		−0.025	0.105		−0.030	0.067		−0.016	0.122		−0.029	0.094
Employment status (unemployed)		−0.280^***^	0.105		−0.272^***^	0.154		−0.246^***^	0.235		−0.268^***^	0.177		−0.248^***^	0.321^**^		−0.244^***^	0.255^*^
Income (50,000–100,000 yuan per year)		−0.073	−0.219		−0.075	−0.242		−0.085^*^	−0.322^**^		−0.094^**^	−0.322^**^		−0.062	−0.206		−0.099^**^	−0.348^**^
Income (more than 100,000 yuan per year)		0.041	0.050		0.045	0.042		0.037	−0.089		0.026	−0.088		0.045	0.033		0.007	−0.124
Hukou (non-local)		0.020	−0.165		0.033	−0.100		0.046	−0.031		0.037	−0.038		0.053	−0.050		0.071	−0.001
Health (relatively healthy)		−0.351^***^	−0.148		−0.346^***^	−0.106		−0.324^***^	−0.078		−0.338^***^	−0.113		−0.327^***^	−0.036		−0.331^***^	−0.072
Health (neutral)		−0.252^***^	0.191		−0.248^***^	0.236		−0.220^***^	0.275		−0.241^***^	0.223		−0.226^***^	0.336		−0.232^***^	0.296
Health (relatively unhealthy)		−0.009	0.427		0.000	0.518^*^		0.026	0.447		0.004	0.358		0.022	0.606^**^		0.019	0.485^*^
Health (unhealthy)		0.008	1.116^***^		0.000	1.043^**^		0.024	0.669^*^		0.013	0.701^*^		0.012	0.878^**^		0.001	0.665^*^
Relative income (nearly)		0.225^***^	0.033		0.224^***^	0.021		0.232^***^	0.010		0.229^***^	0.039		0.238^***^	−0.046		0.234^***^	−0.040
Relative income (lower)		0.159^***^	−0.317		0.158^***^	−0.294		0.152^**^	−0.375^*^		0.151^**^	−0.394^**^		0.156^***^	−0.430^**^		0.147^**^	−0.426^**^
Living condition (along or with spouse)		1.283^***^	2.631^***^		1.383^***^	3.143^***^		1.422^***^	3.218^***^		1.322^***^	2.988^***^		1.505^***^	3.799^***^		1.538^***^	3.453^***^
Exercise times		0.013	−0.047		0.011	−0.068		0.008	−0.093		0.014	−0.076		0.003	−0.101^*^		0.007	−0.094
SAT × Probability_air_			−0.224			0.023			0.258			0.156			9.242			0.211
SAT × pollutants concentration decline rate			−0.233^***^			0.150^***^			−0.005			0.046^***^			−0.264^***^			0.051^***^
SAT × population			−0.001^***^			−0.001^***^			−0.001^***^			−0.001^***^			−0.001^***^			−0.001^***^
SAT × GDP			0.000^***^			0.000^***^			0.000^***^			0.000^***^			0.000^***^			0.000^***^
SAT × housing price/income			−0.476^**^			−0.855^***^			−1.252^***^			−1.176^***^			−1.241^***^			−1.266^***^
SAT × property (rental)			0.323^***^			0.323^***^			0.303^***^			0.298^***^			0.307^***^			0.301^***^
SAT × living time (5–10 years)			−0.342^***^			−0.337^***^			−0.314^***^			−0.323^***^			−0.335^***^			−0.316^***^
SAT × living time (more than 10 years)			−0.204^***^			−0.198^***^			−0.204^***^			−0.222^***^			−0.179^***^			−0.199^***^
SAT × gender (female)			−0.050			−0.048			−0.038			−0.029			−0.042			−0.037
SAT × employment (unemployed)			−0.113^***^			−0.125^***^			−0.142^***^			−0.132^***^			−0.163^***^			−0.148^***^
SAT × income (50,000–100,000 yuan per year)			0.039			0.044			0.063			0.061			0.038			0.067
SAT × income (more than 100,000 yuan per year)			0.008			0.011			0.043			0.039			0.015			0.044
SAT × hukou (non-local)			0.060			0.044			0.030			0.031			0.035			0.028
SAT × health (relatively healthy)			−0.043			−0.053			−0.059			−0.055			−0.069			−0.062
SAT × health (neutral)			−0.108^**^			−0.119^**^			−0.129^**^			−0.121^**^			−0.143^***^			−0.137^***^
SAT × health (relatively unhealthy)			−0.096			−0.118			−0.102			−0.084			−0.140^*^			−0.114
SAT × health (unhealthy)			−0.291^**^			−0.274^**^			−0.178			−0.190			−0.226^*^			−0.182
SAT × relative income (nearly)			0.061			0.064			0.069			0.060			0.084			0.083
SAT × relative income (lower)			0.135^**^			0.130^**^			0.152^***^			0.156^***^			0.166^***^			0.165^***^
SAT × living condition (along or with spouse)			−0.403^***^			−0.523^***^			−0.542^***^			−0.503^***^			−0.680^***^			−0.577^***^
SAT × exercise times			0.018			0.023			0.028^*^			0.025			0.030^*^			0.028^*^
Constant	0.552^***^	0.931^***^	1.249^***^	0.552^***^	0.805^***^	0.420	0.552^***^	−0.015	−2.202^***^	0.552^***^	0.415^***^	−1.357^***^	0.552^***^	0.423^***^	−1.155^***^	0.552^***^	0.150	−1.931^***^

^***^p < 0.01; ^**^p < 0.05; ^*^p < 0.1.

Model 6 reveals that exposure all the pollutants still significantly predicted higher relocation propensity. At the urban scale, the spatially weighted differences in PM_2.5_ and PM_10_ levels between cities enhance the willingness of older adults to relocate. Furthermore, the pollutants concentration decline rate is negatively correlated with the relocation willingness of older adults, indicating that as cities strengthen their efforts to combat air pollution, the willingness of older adult individuals to move can be reduced. At the individual level, older adults who are renting, have shorter residence durations, are employed, are in better health, have relatively lower income, and either live alone or with a spouse tend to have a higher willingness to relocate.

Model 7 identifies significant moderation effects. With the inclusion of moderating variables, the negative impact of satisfaction on the relocation intentions of older adult individuals has weakened for NO_2_, SO_2_, CO, and O_3_. However, the overall relationship remains significantly negative. In addition to the spatially weighted differences in pollutant concentrations and living conditions, the significance and correlation of all moderating variables are generally consistent with their regression results when treated as control variables in Model 6. This suggests that at the urban scale, stronger efforts in air pollution control and a better urban environment, characterized by lower population density, reduced housing prices, and higher economic levels, can mitigate the negative correlation between satisfaction and the relocation willingness of older adults.

At the individual level, unstable living conditions, such as shorter residency duration, lack of property ownership, and being non-retired, as well as better health and lower relative income, also contribute to a reduction in the negative correlation between satisfaction and relocation willingness among the older adult. Combining the insights from Models 3 and 4, it is evident that these influencing factors predominantly pertain to older adults who are in good health, possess a certain degree of labor capacity, and are still engaged in productive work. These individuals may experience job instability while maintaining higher levels of satisfaction. Furthermore, consistent with our hypothesis, living alone or cohabiting with a spouse may exacerbate the negative correlation between satisfaction and relocation willingness among the older adult.

### 4.3 Endogeneity test

Due to the potential for reverse causality between older adults' perceptions of air pollution and their willingness to relocate, this study employs instrumental variable approach to address the impact of this endogeneity. The chosen instrumental variables must be correlated with the explanatory variable, namely the satisfaction of older adult individuals regarding air quality, while being uncorrelated with the random error term. Based on the regression results from Models 3–6, this study selects gender as the instrumental variable for the endogeneity test.

The results from the first stage of Table 5 indicate that the regression coefficient for gender as an instrumental variable is positive and significant at the 1% level, with the *F*-statistic far exceeding 10. This suggests that the choice of gender as an instrumental variable is valid. The results from the second stage of the regression analysis reveal that satisfaction continues to exhibit a significant negative effect on the relocation willingness of older adults, indicating that the baseline conclusions remain valid even after accounting for potential endogeneity issues.

**Table 5:** Estimated results of instrumental variables.

**Variable**	**First stage**	**Second stage**	***F***	***p*-Value**
	**(1)**	**(2)**		
Female	−0.077^***^		45.656	0.031
SAT		−0.070^***^		

In consideration of the readability of the results, only the regression outcomes for the instrumental variables are presented here. ^***^p < 0.01; ^**^p < 0.05; ^*^p < 0.1.

### 4.4 Robust test

Considering that the number of older adults with a willingness to relocate is relatively small, this study employs a subsample analysis to conduct the robustness test. Gender is used as the criterion for classifying the subsamples. Table 6 presents the results of the robust test for the subsamples. The results indicate that for both male and female older adults, their satisfaction with air quality continues to exhibit a significant negative correlation with their willingness to relocate. Therefore, it can be reasonably assured that the original model's robustness is upheld.

**Table 6:** Estimated results of robust test.

**Variable**	**Subsample**	**Coefficient**
SAT	Male	−0.827^***^
	Female	−0.909^***^

In consideration of the readability of the results, only the regression outcomes for the gender variables are presented here. ^***^p < 0.01; ^**^p < 0.05; ^*^p < 0.1.

## 5 Discussion

This study investigates how air pollution perceptions shape relocation intentions among older adult populations in China. The key finding—that air quality dissatisfaction significantly predicts relocation willingness in heavily polluted cities—aligns with existing evidence on pollution-induced health risks for older adults. Importantly, our two-stage analytical framework advances prior work by disentangling the dual mechanisms through which air pollution influences migration decisions: (1) direct environmental push factors and (2) perception-mediated psychological pathways. This mediation effect, quantified through regression analysis, reveals that urban characteristics (economic development level, population density, and environmental governance capacity) and individual attributes (income, housing conditions) jointly shape older adults' health risk perceptions, which subsequently drive relocation considerations.

Specially, air quality satisfaction serves as a critical mediator between different air pollutants and relocation willingness. Regarding the concentration indicators of four types of gaseous pollutants, including PM_2.5_, NO_2_, SO_2_, and CO, older adults' perceptions of air quality serve as a mediating factor that exacerbates the relationship between atmospheric pollutant concentrations and residents' willingness to relocate while PM_2.5_ exhibits paradoxical effects which directly suppressing relocation. As discussed in the theoretical framework, for certain pollutants, the perception of air quality may play a bidirectional mediating role in the relationship between atmospheric pollution and the willingness of older adult individuals to relocate. PM_2.5_ has been the most prominently mentioned air pollution indicator in Chinese media over the past decade and has been a key target in the Chinese government's pollution control efforts. An environment with high PM_2.5_ levels, coupled with excessive media promotion, may lead to a desensitization effect in older adult individuals regarding their perception of air pollution, from both medical and psychological perspectives.

These findings highlight the environmental inequalities faced by some older adult individuals, as well as the social constraints that contribute to their situation. Socioeconomically disadvantaged older adults in less regulated cities may develop attenuated pollution risk perceptions due to limited health literacy and adaptive resources that perpetuates their exposure to hazardous environments. Moreover, air pollution exacerbates the incidence of chronic diseases among the older adult (49). Those older adults who are relatively healthy may not perceive these gradual effects, resulting in prolonged exposure to atmospheric pollution. Furthermore, the empirical findings of this study partially confirm our theoretical hypotheses. Prolonged exposure to air pollution among the older adult reduces their evaluation of air quality; however, the deep-rooted emotional attachment to their long-term residences—encompassing interpersonal relationships and lifestyle habits—hinders their ability to leave polluted environments. Additionally, China's unique intergenerational dynamics play a significant role, as older adults living with their children are less likely to relocate due to the responsibilities of caring for their grandchildren. Such implicit inequalities, often overlooked in urban planning paradigms, demand urgent policy attention given China's rapidly aging population and spatially uneven pollution control measures.

Building on China's “Healthy Aging 2030” initiative and the Ecological Civilization policy framework, we propose three targeted interventions informed by our findings. First, targeted air quality education programs should be implemented to enhance health literacy among older adults, focusing on the risks associated with air pollution and practical mitigation strategies, particularly in heavily polluted cities. These programs should focus on enhancing health literacy by disseminating clear, accessible information about the health risks associated with air pollution and practical measures to mitigate exposure. Collaborating with local healthcare providers and non-governmental organizations (NGOs) to deliver workshops and seminars that address the specific needs of older adults, including those with chronic illnesses or limited mobility, can also raise awareness among the older adult about air pollution. Additionally, establishing subsidized relocation programs can provide financial support for older adult residents in heavily polluted areas, facilitating their transition to healthier environments. Integrating health interventions with air quality initiatives, such as free health screenings, can further support vulnerable populations. Public awareness campaigns aimed at raising awareness of the long-term health risks of air pollution, particularly among older adult individuals and their families, are also essential. Finally, policies should consider intergenerational dynamics by offering support for older adult caregivers, ensuring that family responsibilities do not hinder relocation opportunities, such as offering childcare assistance or flexible housing solutions that accommodate multigenerational households or encouraging local governments to develop family-friendly policies that address the needs of older adult caregivers.

Despite its contributions, this study has several limitations that warrant consideration. Due to data limitations, this study primarily focuses on the relationship between intercity air pollution and the relocation intentions of the older adult at a national scale, without accounting for intra-city variations, such as differences between urban and rural areas, as well as disparities among communities. We hope that future research can further investigate the perceptions of air pollution and relocation responses among different older adult populations at a more micro-level scale. The cross-sectional design limits causal inference, suggesting the need for longitudinal studies to track changes over time. Additionally, reliance on self-reported data may introduce biases; thus, incorporating objective measures of air quality and behaviors in future studies is recommended. The findings may also not fully capture cultural and regional variations in attitudes toward pollution and relocation. Lastly, while the study suggests several policies, it does not address the practical challenges of implementing these recommendations, indicating a need for future research to evaluate their feasibility through pilot programs.

## 6 Conclusions

In conclusion, this study provides valuable insights into the intricate relationship between air pollution perceptions and relocation intentions among older adult populations in China. Our findings underscore the significant role of air quality dissatisfaction as a predictor of relocation willingness in heavily polluted urban areas, highlighting the multifaceted nature of this issue. By employing a two-stage analytical framework, we have elucidated the direct environmental push factors and the perception-mediated psychological pathways that influence migration decisions. The results reveal that urban characteristics and individual attributes collectively shape older adults' health risk perceptions, which in turn inform their relocation considerations. Notably, the mediating role of air quality satisfaction between various air pollutants and relocation willingness emphasizes the need for targeted interventions that address both environmental and psychological dimensions of air quality. Given the pressing challenges posed by air pollution and its disproportionate impact on vulnerable older adult populations, our study calls for urgent policy measures that prioritize health literacy, subsidized relocation programs, and intergenerational support systems. These interventions are essential for fostering healthier living environments and enhancing the overall wellbeing of older adults.

While this study contributes to the understanding of air pollution's effects on older adult populations, it also highlights several limitations that future research should address. By exploring intra-city variations and employing longitudinal designs, subsequent studies can further refine our understanding of how air pollution perceptions evolve and influence relocation decisions among diverse older adult groups. Ultimately, as China continues to grapple with the dual challenges of an aging population and environmental degradation, it is imperative that policymakers, researchers, and community stakeholders collaborate to create inclusive and effective strategies that mitigate the adverse effects of air pollution on the older adult, ensuring a healthier and more equitable future for all.

## Data Availability

The data analyzed in this study is subject to the following licenses/restrictions. Data available on request due to policy restriction. Requests to access these datasets should be directed to xiongzhifei2019@igsnrr.ac.cn.

## Appendix 1

Questionnaire 1.

1. Do you think urban (where you live in) air pollution are serious:

A. Light (very satisfied) B. Relatively light (Satisfied) C. Neutral D. relatively serious (dissatisfied) E. Serious (very dissatisfied)

2. What is the property ownership of your current house:

A. Ownership B. Rental (Including public rental house)

3. How long have you lived in your current residence:

A. Less than 1 year B. 1 to 2 years C. 3 to 4 years D. 5 to 9 years E. More than 10 years

4. What is your gender:

A. Male B. Female

5. Are you currently employed:

A. Yes B. No

6. What is your family's annual total income:

A. Less than 50,000 yuan B. 50,000 to 99,999 yuan C. 100,000 to 199,999 yuan D. 200,000 to 299,999 yuan E. 300,000 to 499,999 yuan F. More than 500,000

7. What is your registered residence (Hukou):

A. Born locally, with local household registration B. Out of town household registration (non-local) C. Born out of town, obtained local household registration upon reaching adulthood

8. Compared to the people you know around you, your income:

A. Higher B. Nearly C. Lower

9. Do you think your physical health condition is good:

A. Healthy B. Relatively healthy C. Neutral D. Relatively unhealthy E. Unhealthy

10. Do you have any plans to relocate in the next 3 years:

A. Yes B. No

11. How many times do you exercise per week _____

12. The number of family members living together _____ and their relationship to you _____

A. Grandparents B. Parents C. Spouse D. Children E. Grandchildren F. Siblings G. Others _____
